# Acute pancreatitis due to *Leptospira* spp.: An uncommon case report of a common zoonosis

**DOI:** 10.7705/biomedica.7771

**Published:** 2026-07-22

**Authors:** Juan Pablo López, Carlos Alberto Salgado, Cristhian David Morales

**Affiliations:** 1 Servicio de Hospitalización, Clínica Nueva de Cali, Cali, Colombia Clínica Nueva de Cali Cali Colombia; 2 Servicio de Medicina Interna, Clínica Nueva de Cali, Cali, Colombia Clínica Nueva de Cali Cali Colombia; 3 Programa de Medicina Interna, Pontificia Universidad Javeriana, Cali, Colombia Pontificia Universidad Javeriana Pontificia Universidad Javeriana Cali Colombia; 4 Programa de Especialización en Medicina Forense, Pontificia Universidad Javeriana, Cali, Colombia Pontificia Universidad Javeriana Pontificia Universidad Javeriana Cali Colombia; 5 Departamento de Salud Pública, Facultad Salud, Universidad Autónoma de Manizales, Manizales, Colombia Universidad Autónoma de Manizales Facultad Salud Universidad Autónoma de Manizales Manizales Colombia

**Keywords:** Leptospirosis, pancreatitis, zoonoses, communicable diseases, One Health, public health, leptospirosis, pancreatitis, zoonosis, enfermedades transmisibles, salud única, salud pública

## Abstract

Leptospirosis, a widespread zoonotic disease prevalent in tropical and subtropical regions, causes over one million cases and about 59,000 deaths annually. It is transmitted through contact with infected animal urine, primarily from rodents, and worsened by poor sanitation and favorable climate. Although in Colombia there was a significant decline in cases from 2018 to 2022, severe forms of the disease, including rare complications like acute pancreatitis, still pose significant health risks.

A 49-year-old male presented with symptoms including abdominal pain, jaundice, and severe thrombocytopenia. Despite initial normal tests, leptospirosis was eventually diagnosed after further testing revealed elevated lipase levels and medical imaging confirmed severe pancreatitis. The patient received antibiotics and intensive care, which led to recovery and confirmation of leptospirosis through serology. Leptospirosis can cause severe multi-organ complications, including rare cases of pancreatitis. This case underscores the need for early diagnosis and treatment of leptospirosis to manage such complications effectively. Environmental factors and rodent control are crucial for prevention, emphasizing the importance of comprehensive management and preventive measures.

## Resumen

La leptospirosis es una enfermedad zoonótica extendida y prevalente en regiones tropicales y subtropicales, que causa más de un millón de casos y alrededor de 59.000 muertes anuales. Se transmite por el contacto con la orina de animales infectados, principalmente de roedores, y se agrava por la falta de saneamiento y un clima favorable. Aunque en Colombia se observó una disminución significativa de los casos entre el 2018 y el 2022, las formas graves de la enfermedad, incluidas complicaciones raras como la pancreatitis aguda, siguen representando riesgos importantes para la salud. Un hombre de 49 años presentó dolor abdominal, ictericia y trombocitopenia grave. A pesar de las pruebas iniciales normales, se diagnosticó leptospirosis tras pruebas adicionales que revelaron aumento de la lipasa y la confirmación, mediante imágenes, de una pancreatitis grave. El paciente recibió tratamiento con antibióticos y cuidados intensivos, lo que llevó a su recuperación, y la leptospirosis se confirmó mediante serología. Esta enfermedad puede causar complicaciones graves multiorgánicas, incluyendo casos raros de pancreatitis. Con este caso se subraya la necesidad de un diagnóstico y un tratamiento tempranos de la leptospirosis, para manejar de manera efectiva sus complicaciones. Los factores ambientales y el control de roedores son fundamentales para la prevención, destacándose la importancia de una gestión y medidas preventivas integrales.

Despite significant efforts by various countries and non-governmental organizations, leptospirosis is considered the most widespread zoonosis globally, with particularly high incidence in tropical and subtropical regions ^1^. It is estimated that there are over one million cases and approximately 59,000 deaths worldwide each year, highlighting its relevance to public health, especially in developing countries ^1^. Favorable climatic conditions and inadequate sanitation in these regions contribute to the spread of the disease, which is transmitted through direct or indirect contact with the urine of infected animals, particularly rodents ^1^^,^^2^. According to the Panamerican Health Organization, the prevalence of leptospirosis in Colombia during 2023 was 29%, with 7,818 cases reported by the national public health surveillance system *(Sistema Nacional de Vigilancia en Salud Pública de Colombia,* SIVIGILA) in the same year ^3^.

Leptospirosis presents a wide range of clinical manifestations, from asymptomatic infections or mild febrile episodes to severe forms that can result in multiorgan dysfunction and high mortality ^4^^-^^6^. Severe forms may include jaundice, hemorrhages, renal failure, and severe pulmonary complications, with Weil's syndrome being one of the most lethal forms ^5^. Although rare, leptospirosis can also cause acute pancreatitis, a rare but potentially serious complication ^5^^,^^6^.

Acute pancreatitis in the context of leptospirosis is an uncommon complication, and its incidence, pathogenesis, and risk factors are not well-documented ^5^^-^^7^. Pancreatitis can vary from mild presentations to severe forms with pancreatic necrosis and multiorgan failure ^5^^-^^7^. Diagnosing pancreatitis in patients with leptospirosis is crucial, as both conditions can lead to multiorgan dysfunction, and early diagnosis and treatment can reduce the risk of mortality ^4^^-^^7^. Pancreatitis is diagnosed by correlating biochemical and radiological findings with the patient's clinical presentation ^4^^-^^7^. According to the modified Atlanta criteria, the presence of at least two of the following three criteria is required: clinical characteristics (such as abdominal pain, nausea, and vomiting), biochemical characteristics (serum lipase or amylase concentration three times higher than the normal limit), and characteristic findings on computed tomography (CT) or magnetic resonance imaging (MRI) ^8^.

On the other hand, diagnosing leptospirosis is challenging due to the diversity of its clinical presentations and the overlap of symptoms with other infectious diseases ^6^^,^^7^. Diagnostic tests include the microscopic agglutination test (MAT), although its availability and sensitivity during the acute phases of the disease are limited ^5^. Other diagnostic methods such as ELISA IgM and various rapid tests can assist in detection, but they also have limitations during the early stages of the infection ^5^.

The clinical manifestations of leptospirosis and acute pancreatitis can be similar, complicating the diagnosis ^4^^-^^7^. However, the early identification and treatment of both conditions are essential to minimize complications and improve clinical outcomes ^4^^-^^7^.

In this report, we present a case of leptospirosis that resulted in acute pancreatitis. This case illustrates the importance of considering rare diagnoses and unusual complications in patients with leptospirosis. It underscores the need for comprehensive medical care and timely management to reduce associated morbidity and mortality.

## Case report

A 49-year-old male with no significant past medical history exhibited symptoms that developed over 12 days, characterized by general malaise, asthenia, adynamia, abdominal pain localized in the epigastrium associated with distension and postprandial emesis with oral intolerance, as well as an unquantified fever that resolved spontaneously. He was evaluated and referred on an outpatient basis due to the finding of total hyperbilirubinemia at 20 mg/dl, primarily due to direct bilirubin, accompanied by leukocytosis and severe thrombocytopenia (table 1).

**Table 1:** Laboratory results at admission and discharge, and additional studies performed for differential diagnosis

Laboratory	Detail	Result	At discharge
Complete blood count	Leukocytes (per μl)	8,660	5,200
	Neutrophils (per μl)	7,360	3,090
	Lymphocytes (per μl)	500	1,370
	Hemoglobin (mg/dl)	12.5	7.8
	Hematocrit (mg/dl)	32.9	23.1
	Platelets (per μl)	277,000	526,000
Blood chemistry	Creatinine (mg/dl)	8.76	1.2
	Blood urea nitrogen (mg/dl)	-	12.4
	Sodium (mmol/L)	-	138
	Potassium (mmol/L)	-	3.82
	Chloride (mmol/L)	-	104.5
	Total bilirrubin (mg/dl)	40	3.47
	Direct bilirrubin (mg/dl)	35.82	3.42
	Indirect bilirrubin (mg/dl)	4.18	0.05
	Alkaline phosphatase (U/L)	196	128
	Amylase (mg/dl)	380	-
	Lipase (mg/dl)	1,037.9	-
	Aspartate aminotransferase (U/L)	53	94
	Alanine aminotransferase (U/L)	55	169
Differential diagnoses	Condition	Test performed	Result
	Dengue virus infection	IgM antibodies	Negative
	Human immunodeficiency virus	ELISA	Non-reactive
	Syphilis	Serology	Negative
	Hepatitis B virus infection	Surface antibodies	Non-reactive
	Hepatitis C virus infection	Anti-HCV antibodies	Non-reactive
	Hypertriglyceridemia	Serum triglyceride level	457 mg/dl
	Immunorheumatologic disease suspicion	Antinuclear antibodies titre	1/320; fine granular pattern

Physical examination revealed pronounced generalized jaundice and low-range blood pressure. Abdominal pain was observed upon palpation in the upper hemiabdomen. Consequently, a comprehensive diagnostic approach was undertaken, including serologic testing for dengue viruses, hepatotropic viruses (HBV and HCV), human immunodeficiency virus (HIV) and syphilis, in addition to serum triglyceride measurement and antinuclear antibody (ANA) titration to evaluate for a potential immunorheumatologic etiology (table 1).

An urgent abdominal ultrasound was also performed. Upon reevaluation, the abdominal ultrasound revealed no abnormalities. All infectious serologies returned negative. Although serum triglyceride levels were elevated (457 mg/dl), they remained below the threshold commonly associated with hypertriglyceridemia-induced pancreatitis (≥ 1,000 mg/dl), suggesting this etiology was improbable. Furthermore, the ANA test was positive at a titer of 1:320 with a fine granular pattern -a positive result considered nonspecific-, which, in the absence of corroborating clinical features, was insufficient to establish a rheumatological diagnosis. In addition, to assess exposure factors for potential infections or diseases related to the environment and contact with rodents or stagnant water, we conducted a series of questions to identify the risks of zoonotic infections (table 2). Due to this presentation and some risks of zoonotic infections identified, leptospirosis was suspected. IgG and IgM tests and an abdominal CT scan were ordered, which later ruled out biliary tract involvement.

**Table 2:** Exposure assessment for environmental and zoonotic infection risk factors

Question	Answer
Have you been in contact with stagnant or flowing water in the past few weeks?	No
Do you participate in recreational activities in rivers, ponds, or areas with stagnant water?	No
Do you have any wounds or skin lesions that may have come in contact with contaminated water?	No
Have you had recent contact with rodents or animals that may be infected?	Yes, rodents (rats, mice)
Have you noticed high presence of rodents in your nearby environment?	Yes, although currently under control
Are there rodents in your home or in the area where you frequently spend time?	No
How would you describe the state of the water in your surroundings, for example, stagnant water?	Stagnant water in tires
What is the state of sanitation infrastructure in your area?	Good condition
Are there community efforts to improve wastewater management and waste disposal?	Trash is collected three times a week, so it doesn't remain on the street for long periods.
Do you use protective clothing or special equipment during activities in humid environments?	No, only boots in wet weather and gloves due to being a mechanic.

The patient continued to progress with acute renal failure criteria AKIN III, eventually requiring renal replacement therapy as life support. A lipase report arrived with a level of 1037.9 mg/dl, indicating severe acute pancreatitis. Empirical treatment with piperacillin and tazobactam was initiated, and the patient was transferred to the intensive care unit for continued management. During the intensive care unit stay, and after ruling out infectious, biliary, and metabolic causes, the suspicion of a possible autoimmune phenomenon as the underlying cause was maintained. In this context, a portal Doppler ultrasound was performed to assess associated vascular involvement, such as portal vein thrombosis or signs of portal hypertension. The Doppler study revealed no evidence of thrombosis or hemodynamic alterations.

Due to favorable clinical evolution and after 7 days of intensive care management, the patient experienced gradual recovery of renal function, leading to the removal of the hemodialysis catheter. He was transferred to the general ward, where the results of IgG and IgM antibodies for *Leptospira* spp. came back positive, clarifying the diagnosis of severe leptospirosis complicated by pancreatitis, also meeting severity criteria. He was evaluated by the hepatology service, who deemed it unnecessary to continue autoimmune studies as they identified the infectious origin of the condition. They recommended the completion of the proposed antibiotic regimen and hospital discharge.

### Ethical considerations

Written informed consent was obtained from the patient for the publication of this case for academic purposes

## Discussion

Leptospirosis, caused by spirochetes of the genus *Leptospira,* is a zoonotic disease with a broad spectrum of clinical manifestations ^2^^-^^5^. The pathogenic species, *Leptospira interrogans,* comprises various serogroups and serovars, with *Leptospira icterohaemorrhagiae* commonly associated with severe forms of the disease ^2^^-^^5^. The infection can lead to widespread vasculitis, resulting in damage to multiple organs, including renal, hepatic, hematological, meningeal, pulmonary, myocardial, and, rarely, pancreatic involvement ^4^^-^^7^.

In this case report, the patient exhibited symptoms indicative of severe leptospirosis, involving multiple organs typically affected by the disease ^6^. However, the notable involvement of acute pancreatitis is rare and not well-documented in human leptospirosis ^4^^-^^7^. Acute pancreatitis can be induced by various microorganisms, including bacteria such as *Leptospira* spp. ^4^^-^^7^. Initial associations between pancreatitis and leptospirosis were described in the 1950s ^5^. Subsequent studies have documented pancreatic involvement in leptospirosis cases, though it remains a rare complication ^5^. For instance, a study of 33 autopsied patients who died of leptospirosis found pancreatic involvement in 36.6% of cases, while another study reported pancreatic damage in 13 out of 20 cases ^5^.

Pancreatic lesions in leptospirosis range from edema and inflammatory infiltrates to hemorrhagic and necrotic damage, reflecting a wide spectrum of pancreatic involvement ^4^^-^^7^. The presence of pancreatic necrosis, as identified by contrast-enhanced computed tomography (CT), defines severe pancreatitis and significantly affects prognosis, with mortality rates reaching up to 20% ^8^. However, according to the revised Atlanta classification, imaging is not essential for determining severity in the absence of clinical signs of complications, and its use is primarily reserved for patients with suspected severe disease or clinical deterioration. In our case, the patient presented with classic features of acute pancreatitis, including elevated lipase levels, but without radiological evidence of pancreatic or peripancreatic inflammation or necrosis ^9^. These findings support a mild form of pancreatitis under the Atlanta criteria. Additionally, differential diagnosis excluded biliary causes such as cholelithiasis and choledocholithiasis, which are among the most frequent etiologies of acute pancreatitis ^8^^,^^9^.

Leptospirosis diagnosis was confirmed through serology, which is highly suggestive, in endemic areas ^4^^-^^7^. While the MAT remains the gold standard for leptospirosis diagnosis, PCR may offer earlier detection, facilitating timely antibiotic administration ^5^. Despite being less specific, serological tests like ELISA are faster, more accessible, and useful in the acute phases of infection, while the MAT test, although more precise for identifying *Leptospira* serovars in later stages, requires a specialized laboratory ^5^. The patient responded well to medical treatment, with resolution of clinical symptoms and normalization of laboratory findings. While surgical intervention for pancreatic complications in leptospirosis has been reported, it was not necessary in this case ^10^.

Leptospirosis-associated pancreatitis is rarely reported, with only a few cases documented. The exact pathophysiology remains unclear, but it is hypothesized that the activation of toll-like receptors (TLR2) may trigger inflammatory pathways leading to vasculitis and endothelial damage in the pancreas ^5^. In the reviewed cases, clinical presentations often included typical signs of pancreatitis, such as abdominal pain and elevated pancreatic enzymes, which were also identified in this case ^4^^-^^7^.

This case of pancreatitis illustrates the importance of maintaining a broad differential diagnosis in tropical regions, where acute febrile illnesses with multiorgan involvement may mimic common etiologies such as dengue, viral hepatitis, autoimmune disease, or even HIV ^11^. In our patient, serum triglyceride levels were elevated (457 mg/dl), but remain below the threshold (1000 mg/dl) typically associated with hypertriglyceridemia-induced pancreatitis ^11^^,^^12^. Additionally, antinuclear antibodies (ANA) were weakly positive with a fine granular pattern, a finding that is nonspecific and not uncommon in healthy individuals ^11^. In the absence of clinical signs or organ-specific markers, this result does not support and underlying autoimmune etiology. HIV testing was also negative, helping to rule our immunosuppressive conditions ^11^.

From a One Health perspective, it is essential to consider several factors in the transmission of *Leptospira* spp. ^13^. The patient in this case reported contact with standing water during working activities, which is a well-documented risk factor ^13^. Activities such as swimming in rivers or ponds, particularly in endemic regions, increase the risk of exposure to *Leptospira* spp. through damaged skin or mucous membranes ^13^.

The role of animal reservoirs is fundamental in the epidemiology of leptospirosis ^13^. Rodents are the primary reservoirs due to their high infection rates and the capacity to excrete large quantities of spirochetes in their urine ^13^. In both rural and urban areas, rodent control is a key preventive measure. However, the patient reported observing a high presence of rodents in his environment, which indicates the need for pest control measures ^13^.

Regarding environmental factors, climatic conditions play a crucial role in the persistence and spread of *Leptospira* spp. ^13^. The bacteria can survive in warm, moist environments, and flooding events can significantly increase the risk of leptospirosis outbreaks ^13^. In this case, a recent history of heavy rains in the region was identified, which may have contributed to the contamination of surface waters with *Leptospira* spp.

Prevention strategies should include community education on the use of protective clothing, improvement of sanitation infrastructure, and avoidance of contact with potentially contaminated water, as well as providing the patient with specific instructions on these measures ^13^. Additionally, vaccination of domestic and farm animals can reduce the infection burden in animal reservoirs and, consequently, transmission to humans ^13^.

The following learning points can be concluded from this case:

1. Common diseases can have uncommon presentations. In endemic regions like Colombia, frequent infectious diseases such as leptospirosis may manifest with atypical clinical syndromes, including acute pancreatitis, highlighting the need to go beyond diagnostic heuristics.
2. It is more likely that a single disease produces multiple clinical syndromes than several unrelated diseases occurring simultaneously. This diagnostic principle is crucial in guiding clinical reasoning, especially in resource-limited or high-burden settings.
3. Case reports of rare presentations in prevalent diseases are valuable to clinical education and diagnostic refinement. Sharing such experiences contributes to the recognition of atypical forms and improves early detection, particularly in tropical medicine.
4. A syndromic and One Health approach is essential in tropical areas. Recognizing the interaction between environmental exposure, zoonotic reservoirs, and human health allows earlier suspicion of leptospirosis and its complications.
5. Serologic or laboratory findings must be interpreted in clinical context. Mild hypertriglyceridemia or weakly positive ANA results should not distract clinicians from considering more probable infectious etiologies without supporting clinical features.
